# 

**Published:** 1890-05

**Authors:** 


$2.00 A YEAR IN ADVANCE.

WHOLE NUMBER, CCCXLVI.

New Series.

May, 1890.

VOL. XXIX. No. 10.

LIBRARY
Index
S G O

### BUFFALO MEDICAL AND SURGICAL JOURNAL.

Established 1845 by AUSTIN FLINT, M. D.

**Editors:**

THOS. LOTHROP, M. D. W. W. POTTER, M. D.

**Associate Editors:**

FRANK HAMILTON POTTER, M. D. JAMES WRIGHT PUTNAM, M. D

WILLIAM C. KRAUSS, M. D. JOHN PARMENTER, M. D.

SCRIPTA FERUNT ANNOS

PUBLISHED MONTHLY.

EUROPEAN AGENCY:
TRÜBNER & CO.,
57 AND 59 LUDGATE HILL, LONDON, E. C.

BUFFALO:
1890.

FOREIGN SUBSCRIPTION, $2.50

Entered at the Post-Office at Buffalo, N. Y., as Second-class Mail Matter.

*Times Printing House, Buffalo, N. Y.*

